# Artificial Intelligence-Empowered Mobilization of Assessments in COVID-19-like Pandemics: A Case Study for Early Flattening of the Curve

**DOI:** 10.3390/ijerph17103437

**Published:** 2020-05-14

**Authors:** Murat Simsek, Burak Kantarci

**Affiliations:** School of Electrical Engineering and Computer Science, University of Ottawa, Ottawa, ON K1N 6N5, Canada; murat.simsek@uottawa.ca

**Keywords:** Artificial Intelligence, public health, COVID-19, mobile assessment centers, pandemics, epidemics, self-organizing feature map, neural networks, optimum route planning

## Abstract

The global outbreak of the Coronavirus Disease 2019 (COVID-19) pandemic has uncovered the fragility of healthcare and public health preparedness and planning against epidemics/pandemics. In addition to the medical practice for treatment and immunization, it is vital to have a thorough understanding of community spread phenomena as related research reports 17.9–30.8% confirmed cases to remain asymptomatic. Therefore, an effective assessment strategy is vital to maximize tested population in a short amount of time. This article proposes an Artificial Intelligence (AI)-driven mobilization strategy for mobile assessment agents for epidemics/pandemics. To this end, a self-organizing feature map (SOFM) is trained by using data acquired from past mobile crowdsensing (MCS) campaigns to model mobility patterns of individuals in multiple districts of a city so to maximize the assessed population with minimum agents in the shortest possible time. Through simulation results for a real street map on a mobile crowdsensing simulator and considering the worst case analysis, it is shown that on the 15th day following the first confirmed case in the city under the risk of community spread, AI-enabled mobilization of assessment centers can reduce the unassessed population size down to one fourth of the unassessed population under the case when assessment agents are randomly deployed over the entire city.

## 1. Introduction

With the rise of the COVID-19 outbreak, it has been clear that epidemic and pandemic diseases will continue to threaten the societies in the foreseeable future. Newly identified Severe Acute Respiratory Syndrome Coronavirus 2 (SARS-CoV2) has caused a large number of deaths with more than a million confirmed cases worldwide posing a serious threat to public health [1] and public health systems. Reproduction number ($R_{0}$) of an infectious disease denote its severity for public health as it indicates the average number of individuals that are forecast to catch the same disease once contacted by a single contagious individual. As an example, $R_{0}$ of COVID-19 has been reported to be 1.5 [2].

Major implications of infectious diseases, regardless of them being either epidemic or pandemic, are as follows: first, their impact and fatality rates are significant among vulnerable population such as cardiac patients [3]. Second, from the manageability standpoint, a critical issue in the case of an infectious disease is the hospital surge capacity in a region hit by the disease [4]. Lastly, supply chains become extremely fragile in the case of an epidemic or pandemic situation [5].

In order to cope with the implication of epidemic and/or pandemic situations, proactive and preventive strategies are vital. A thorough investigation for future pandemics were carried out by the authors in [6] by using various preparedness strategies against multiple pandemic scenarios. It is worth to note that these efforts build on massive assessment plans. For instance, to cope with the rising affects of SARS-CoV-2, Taiwan Centers for Disease Control initiated testing on 24 January 2020 for individuals that were found symptomatic or suspected to have been infected [7].

In addition to the legacy approaches and strategies to serve for public health, Artificial Intelligence (AI), more specifically Machine Learning (ML), has been considered as an effective and complementary tool to address various cases threatening public health, particularly infectious and communicable diseases [8]. With this in mind and given a significant portion of the infected individuals can be asymptomatic (e.g., can be up to 30% positive-tested cases for COVID-19 [9]), acquisition of mobility patterns and clusters of residents in certain regions becomes critical to monitor, model and project community spread at large. To achieve this, it would be convenient, at low-cost and feasible to use the concepts of mobile crowdsensing [10]. This article proposes a novel methodology to mobilize assessment centers in the case of an epidemic or pandemic disease so to monitor, model and project the confirmed cases so to help decision makers at different levels of governments make strategies proactively for management and logistics for the public. To this end, the proposed methodology uses the concept of mobile crowdsensing to acquire spatiotemporal mobility data from individuals living and/or commuting in various regions of a city, and applies self-organizing feature maps (SOFM) to deploy mobile assessment centers for pandemic disease in an optimal manner so to achieve minimizing the population that has not been tested with the least possible number of assessment centers in the shortest possible time. The proposal leverages the idea of previously proposed SOFM for threat anticipation in a mobile crowdsensing environment where the objective was maximizing the affected population through malicious tasks/data [11]. Here, the objective is set to minimize the non-covered population in each region given the limited number of mobile assessment centers. Through a pilot study in a real streetmap, numerical results confirm that at the end of the 15th day, SOFM-based deployment and planning of assessment centers can reduce the non-covered (i.e., not tested) population less than one fourth of the case where assessment centers are randomly deployed with given number of destinations per day.

The rest of the article is organized as follows. In Section 2, we present the background and motivation for this study. Section 3 presents the proposed SOFM-based deployment and planning of mobile assessment centers for a pandemic disease. Section 4 presents extensive numerical results simulated on the street map of a real city. Finally, Section 5 concludes the article and provides future directions and open issues.

## 2. Background and Motivation

Recent research shows that pandemic crisis can be coped with preparedness and planning policies as analyzed by the authors in [12] in terms of cost benefits under different preparedness models. Indeed, management of a pandemic crisis also involves planning the assessments of suspicious (i.e., symptomatic) cases as suggested by the authors in [13] based upon the lessons learned from the most recent COVID-19 outbreak. Recently, not to replace entire practice but to complement the existing expertise in public health, empowering Artificial Intelligence (AI) has been considered by several researchers [14]. A comprehensive review of the use of AI in healthcare was carried out by the authors in [15] primarily focusing on issues related to infection cases, more specifically their diagnosis and transmission, as well as, response to treatment and resistance against infection.

Indeed, supporting healthcare and public health system responses to pandemic cases has many aspects varying from modeling of outbreaks to decisions concerning logistics and mobilization. For instance, the authors in [16] focused on pandemics and proposed an intelligent agent-based modeling for the outbreaks to assist the healthcare systems. Another area that can take benefit of AI-based methods is the detection of outbreaks before leading to epidemics and pandemics so to achieve a data-driven monitoring of infectious diseases along with reliable projections [17]. To this end, the authors in [18] proposed a graph structure learning model from unlabeled data. As modeling of epidemics has drawn attention of the researchers in statistics and data science, the study in [19] deployed three different machine learning models, namely back-propagation neural network (BPNN), gradient boosting and random forest (RF) to estimate the probability of Zika outbreak at the global scale, and have shown that the BPNN-based modeling could achieve the highest prediction performance in terms of area under the curve metric. In another study [20], the authors used Recurrent Neural Networks (RNNs) to build prediction models for an outbreak of hand foot mouth disease, and achieved >80% area under the curve prediction performance.

As community spread is one of the major causes of rising cases in epidemics and pandemics, modeling social contact and community behavior is of paramount importance. To this end, the authors in [21] used data from open and commercial sources and proposed a new method to model social contact network in Delhi with the objective of planning for a pandemic outbreak. Modeling of social contact and community behavior can assist in planning of resources in healthcare and public health systems. With these in mind, the authors in [22] proposed the use of Wiener series-based ML to ensure public health services achieve minimum error and optimal resource management targets under the peak situations of an outbreak in terms of spreading speed and the number of accumulated cases.

Based on the state of the art in AI-assisted planning and preparedness against an epidemic/pandemic outbreak, it can be said that one of the major missing components is the mobilization of assessments as the community spread through asymptomatic positive cases, which can vary from 17.9% to 30.8% [9,23]. Based on these facts, the motivation for our study in this article is the gap between monitoring/modeling of the spread and planning of logistic response to epidemic/pandemic outbreak. To this end, we empower an AI-driven strategy to mobilize the assessments in COVID-19-like pandemics.

## 3. AI-Empowered Preparedness for Pandemic Outbreaks

**Figure 1:**
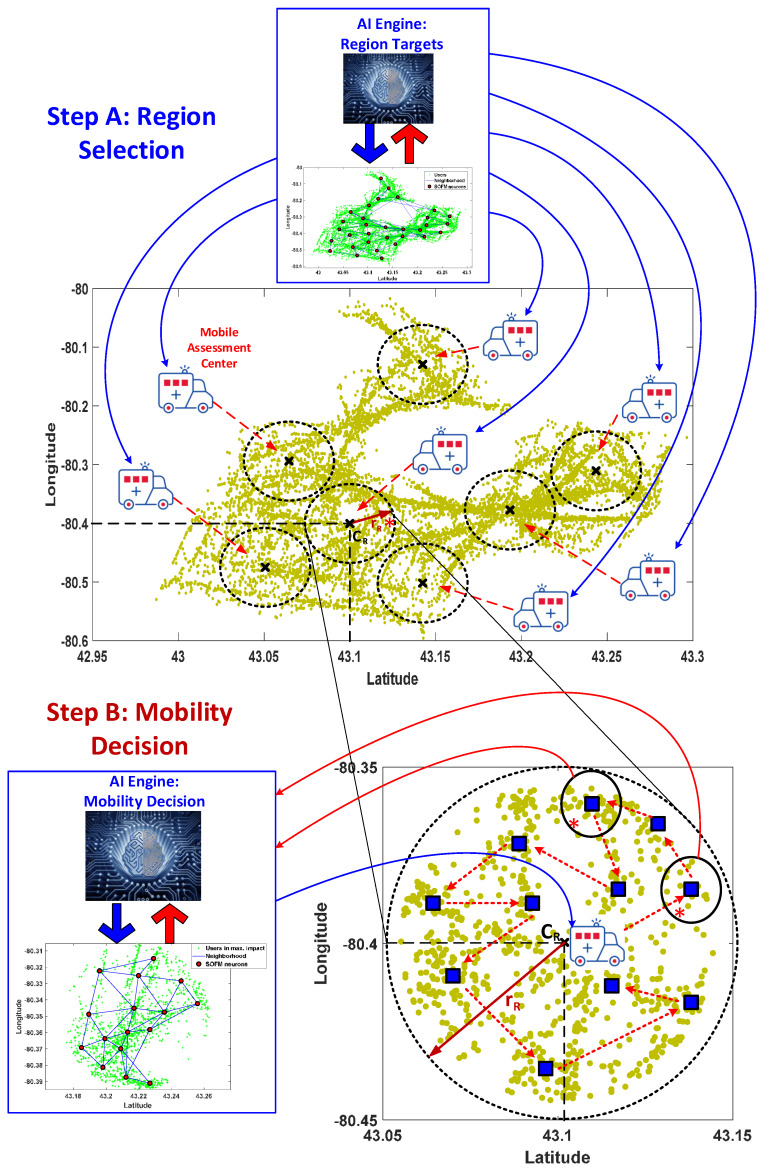
General framework of Artificial Intelligence (AI)-based deployment and route planning decision for mobile assessment center. Step A is self organizing feature map (SOFM)-based region selection and Step B is SOFM-based mobility decision.

Artificial Intelligence (AI) has come forward to design the more intelligent solutions for human’s lives. The health care mostly utilizes key developments in AI world to pioneer in public health. In this work, self organizing feature map (SOFM) is used for the aim of preparedness for pandemic outbreaks as depicted in Figure 1. The pandemic new corona virus COVID-19 is the most vital urgency for all over the world through its rapid spreading characteristic without any symptoms. Preparedness for all pandemics is so critical to flatten the exponential spreading speed of these pandemics.

Mobile assessments are considered to detect infected individuals before they transfer the virus to more individuals to boosting the spreading capacity in this region. SOFM-based AI engine is utilized twice during the process. In the first step (i.e., Step A), the AI engine calls SOFM to determine all possible regions which are defined by the neurons. In the second step (i.e., Step B), SOFM is called to determine the stops for the mobile assessment centers each of which is assigned to serve a single region in the monitored area. In this study, SOFM performance is evaluated in comparison to a random- and uniform grid-based selection policies.

### 3.1. Step A: Region Selection

**Figure 2:**
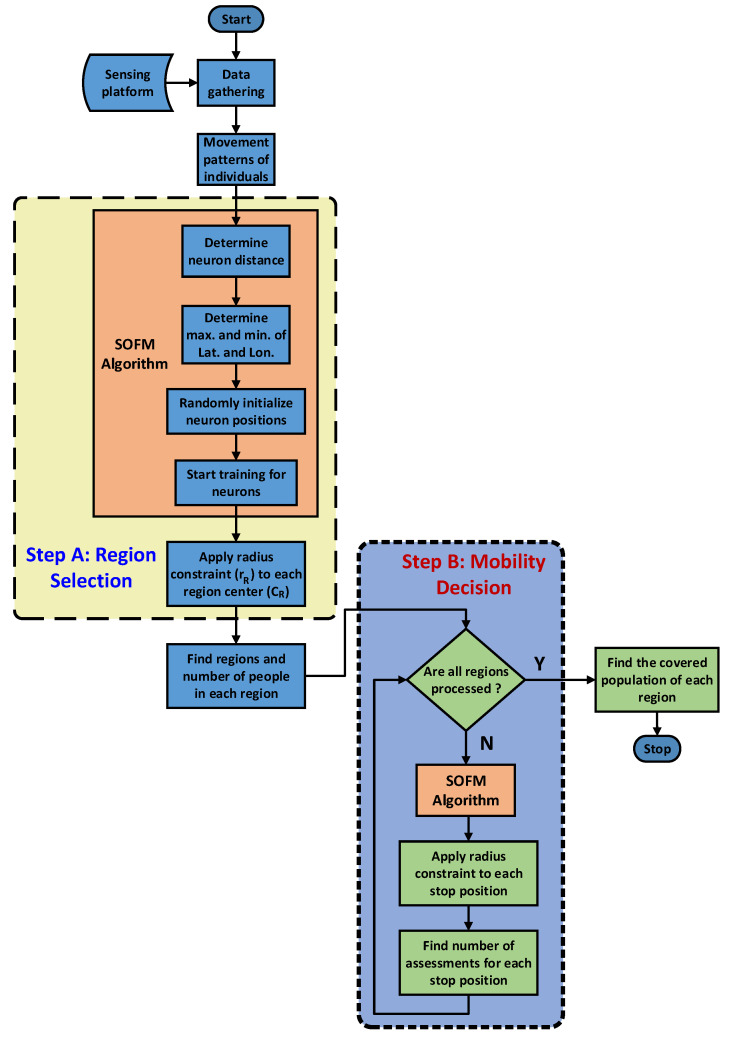
General framework for AI-based solution through SOFM that is used twice in both Steps A and B.

The first step run by the AI engine involves determining the regions for the mobile assessment centers as depicted in Figure 1. The AI engine’s Region Targets module determines the number of neurons by considering the number of mobile assessment centers, the number of population of the deserved area, the target timeline in terms of total number of days to complete the assessments and the availability of mobile assessment centers. In this work, reasonable number of neurons is chosen to verify the efficiency of the proposed method. The area that is monitored due to a priori information about its risk and vulnerability against the pandemic is divided into regions where each region will be assigned a mobile assessment centers. Following upon the training of SOFM, a radius constraint is applied to each region so to determine the number of individuals per region that are marked to be assessed as illustrated in the step A in Figure 2. Regions can be obtained from neuron positions of SOFM such as latitude and longitude. SOFM covers all individuals in the selected area, and following upon applying radius constraint, the coverage can slightly reduce. It is worth to note that covering a set of individuals denotes a set of individuals being marked to be assessed in that region. Considering the worst case scenario to detect all positive (i.e., infected) cases as early as possible, the region that covers the maximum number of people is fed into Step B.

### 3.2. Step B: Mobility Decision

Once the regions have been determined by the Region Targets module of the AI engine, the Mobility Decision module of the AI engine calls SOFM for each region in Step B as illustrated in Figure 1. The objective of the AI engine is two-fold: (1) obtaining all possible stops for each mobile assessment center, and (2) ordering the stops in the each region with respect to the covered population of the each stop. Therefore, detection of the positive cases (i.e., infected individuals) due to the community spread can be achieved faster than the random-based and uniform grid-based selections of the regions and the stops for the selected area. This second step is also highlighted in Figure 2. The number of stops for a mobile assessment center is determined by the number of neurons in the SOFM which is chosen based upon the population size, desired coverage and the target timeline to visit all stops for assessment. In this study, three different numbers of neurons are considered to demonstrate the performance under the worst case situation and the total number of covered population at the end of the assessment process.

### 3.3. Self Organizing Feature Map for Route Planning

SOFM is an unsupervised learning method [24,25,26] for clustering multi-dimensional data into two-dimensional neurons which are directly connected with each other through a pre-defined topology. SOFM is based on competitive learning, similar to winner-take-all networks. The additional feature of SOFM different than winner-take-all is the winner neuron, also called the Best Matching Unit (BMU). BMU is not only updated to be closer to the input coordinates, but also connected neurons of the BMU are updated as well. Therefore BMU and its neighbors are closer to the input coordinates than the other neurons. Here, clustering implies that each neuron in SOFM can cover a set of individuals. Thus, the number of clusters is equal to the number of neurons in SOFM. After applying constraints to SOFM, the population size can be found in that region which is covered by the corresponding SOFM neuron.

This process is repeated for all inputs for each epoch, and finally, the maximum number of epochs is used as the stopping condition for SOFM.

**Figure 3:**
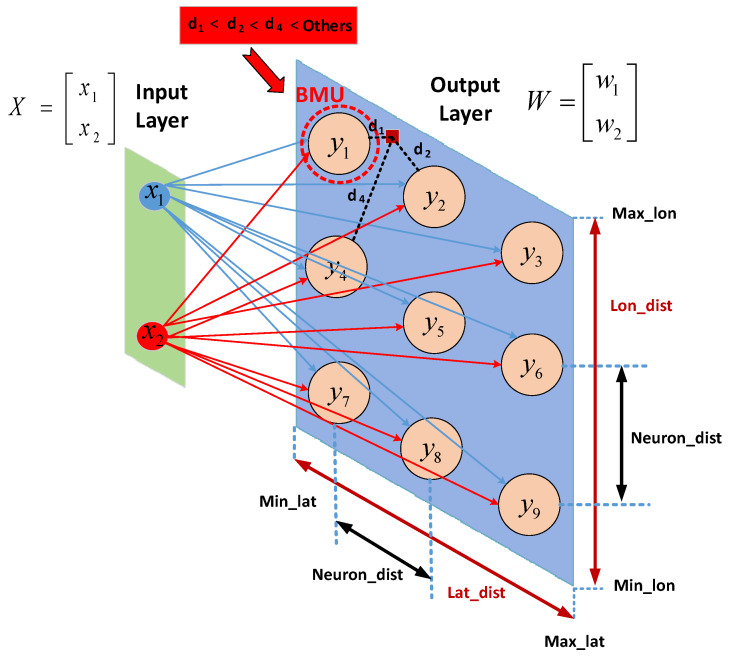
SOFM general structure for locally adaptive number of neurons.

The number of neurons are matched with number of regions in selected area for this study. That is why determining of this neuron number is calculated in terms of two criteria. The first criterion is based on both latitude and longitude intervals of the selected area and second one is directly effected from a uniform grid-based distribution of these neurons. The uniform grid-based distribution is assumed at the beginning of the process about how to find the total number of neurons in order to calculate how many number of neurons are required for latitude axis and longitude axis. The aim of this process is to find the number of neurons according to geometric shape of the selected area. After choosing the neuron distance parameter in Equations (1) and (2) as depicted in Figure 3, each neuron is far from not only other neurons but also minimum and maximum of each coordinate. Neuron numbers for latitude axis in Equation (1) and longitude axis in Equation (2) are multiplied by each other to find the total number of neurons before initialization of each neuron in SOFM training process. The neuron distance parameter Neuron_dist can be chosen as coordinate unit only.

(1) $$Lat\_neuron\_num=round\left\{\frac{Lat\_dist}{Neuron\_dist}\right\}$$

(2) $$Lon\_neuron\_num=round\left\{\frac{Lon\_dist}{Neuron\_dist}\right\}$$

Random initialization for SOFM neurons is selected to initialize neuron positions which place along inside the Lat_dist and Lat_dist as depicted in Figure 3. The training process in SOFM is very similar to the winner-take-all algorithm, which utilizes vectorial distance-based competitive learning. Once each training sample is compared to the weights of each neuron on the 2D hexagonal structure, the Euclidean distance coming out of each comparison is computed separately. As seen in Figure 3, neuron $y_{1}$ can be chosen as BMU through its Euclidean distance less than others for a input coordinate (Lat, Lon) which is depicted as a red square in Figure 3.

The weights of the BMU and its neighboring neurons in the SOFM 2D-grid are adjusted to be closer to the input vector. The magnitude of the change decreases with both time and the distance from the BMU. Distance function for 2 dimensional inputs (Lat, Lon) and weight vectors can be formulated as shown in Equation (3).

(3) $$Dist\left(X,W\right)=\sqrt{\left[\begin{array}{c} x_{lat}-w_{1}\\ x_{lon}-w_{2} \end{array}\right]\times \left[x_{lat}-w_{1},\;x_{lon}-w_{2}\right]}$$

where *X* is the current 2D input vector and *W* is 2D node’s weight vector as shown in Figure 3. Dist is utilized to determine how close *w* is to *x* leading to the information about the output layer neuron that is the best to represent the input features.

(4) $$BMU_{X}=\underset{w}{argmin}∥Dist(X,W^{j})∥\;\mathrm{for}\;j=1,\ldots ,m$$

where BMU indicates best matching neuron *y* to the input *x* as shown in Figure 3.

(5) $$W^{i}=BMU_{X^{i}}$$

where *W* indicates BMU for *i*th input *X*. During the learning phase of SOFM, the area of the neighborhood that is represented by the radius in Equation (6) shrinks according to the number of epochs. Here, $\sigma_{0}$ denotes the initial value of radius and λ denotes a time constant while *i* is the current time-step.

(6) $$\sigma \left(i\right)=\sigma_{0}e^{\left(-\frac{i}{\lambda }\right)}\;i=1,2,3,\ldots Max\_epoch$$

(7) $$W^{\left(i+1\right)}=W^{i}+\Phi \left(i\right)L\left(i\right)\left(X^{i}-W^{i}\right)$$

(8) $$\Phi \left(i\right)=e^{\left(-\frac{Dist^{2}}{2\sigma^{2}\left(i\right)}\right)}$$

(9) $$L\left(i\right)=L_{0}e^{\left(-\frac{i}{\lambda }\right)}$$

where Dist which is the distance from a node to the BMU can be calculated from Equations (3) and σ, is the width of the neighborhood function as calculated by Equation (6). Weights are updated using Equations (7)–(9). Learning rate in Equation (9) is adaptively changed by the iteration steps of SOFM and reduced gradually starting from $L_{0}$.

After the training phase of SOFM, each neuron position is used for the center of each region as the first usage of SOFM technique in the general methodology as indicated in Figure 1 and Figure 2. Normally, SOFM covers 100% to all individuals through neuron clusters. After applying the radius constraint to each neuron used for representing the region of a mobile assessment center, distance-based coverage can be calculated and used for performance metric for comparing with random deployment-based region selection.

SOFM neurons also provide stops for route planning during the testing process to all individuals as fast as possible to detect any infected case in the early-stage before spreading to all healthy regions. Therefore, it is possible to isolate the infected regions from other regions without having infected cases.

## 4. Simulation Settings for COVID-19

Performance evaluation is based on the detection capacity of SOFM-based proposed method in comparison to the random deployment approach. The impact of a single infected case in the region and spreading speed of pandemic are evaluated by using the COVID-19 Simulator [27] designed by Cleve Moller in MATLAB environment.

Crowdsensim simulator [28] is used for generating individuals’ mobility patterns and COVID-19 simulator [27] to generate the spread process following upon the first infected case in a region. The regions are determined by SOFM in the AI engine for Brant with the objective of covering the highest possible non-assessed population day by day given the predicted infections. A total of 1566 individuals are the highest covered population in the region according to other regions. The worst-case scenario is applicable for only the largest population where a single COVID-19 case will be hardly detected by a single mobile assessment center.

By using the COVID-19 simulator, it is possible to show how the disease is spread, and we assume the worst case scenario following upon the first case (i.e., case-0) in a region in order to reach the infected cases. The time passes until the size of population (estimated) becomes less than the number of non-assessed population is critical for COVID-19 community spread detection. Normally detection can be achieved earlier than this worst case scenario. Seven controllable parameters, along with their initial values and the four types of individuals in the COVID-19 simulator are depicted in Table 1.

In the simulations, the number of individuals is chosen as 1566 which equals to maximum number of individuals for applying 5 km radius constraint to all SOFM neurons. The epidemic starts in the upper right quadrant, and ends after all infected individuals have become immune. Individuals are modeled such that one is infected while 80% of the population is mobile and the rest is static. Other parameters in the simulation are chosen as default as depicted in Table 1.

Table 2 presents 30-day results by increasing the number of infected individuals starting with one infected case. Creating the worst case scenario is considered the best way to reveal the efficiency and boundaries of the proposed method to protect the community from the pandemic. According to worst case scenario, three different number of stops for mobile assessment centers are considered, and all of them can detect infected individuals before 30 days; hence the simulation result are presented for up to 30 days in Table 2.

## 5. Numerical Results

**Figure 4:**
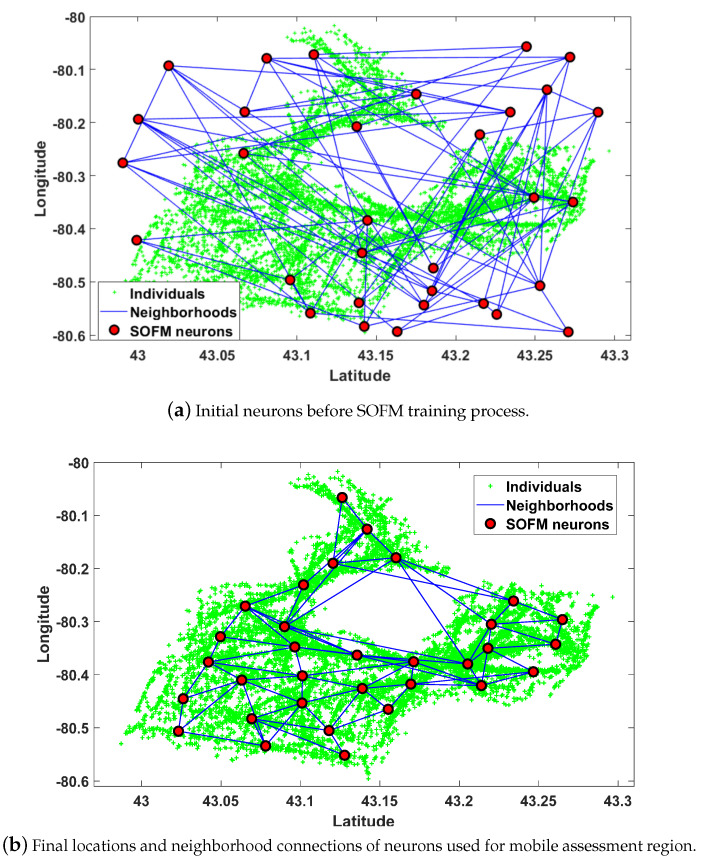
Results of SOFM with the region based adaptive number of neurons represent regions in Brant for mobile assessment centers assignments.

Simulation results for the verification of the proposed approach are evaluated in MATLAB where SOFM functions and additional procedures for analyses are generated. Selected area and the number of individuals are Brant and 10,000, respectively in this article. Brant, which is a city in Ontario, has a population of 36,707 over an area of 843.2 km${}^{2}$. These are obtained from a mobile crowdsensing simulator, Crowdsensim [28]. Since COVID-19 like pandemics are spread by non-symptomatic carrying individuals to other healthy individuals, the fast assessment process is more vital for mobile individuals than static individuals. In this study, 10,000 individuals are used as input for the SOFM algorithm and initial and final locations of neurons representing the center of each region for a single mobile assessment center and also neighborhood connections of each case can be seen in Figure 4. It is worth noting that the number of neurons is one of the parameters of the training process. Therefore, the number of neurons should be chosen according to the population size and the size of the region. In this work, the initial distance is selected to cover all active areas uniformly so the number of neurons can be determined in terms of latitude and longitude intervals and the selected initial distance. The number of neurons are obtained as 32 through the adaptive approach according to the neuron distance at 0.07 as depicted as Figure 3. The 32 neurons are found through 4 (latitude axis) times 8 (longitude axis) according to geographical area of the mobility pattern of individuals. The 2D coordinates of 10,000 individuals which is obtained by Crowdsensim simulator are utilized to determine the active area in terms of upper and lower limits of latitude and longitude. As the neuron distance defines the distance between each neuron for a uniform grid distribution, the active region for SOFM can be uniformly covered according to latitude distance and longitude distance. Since the longitude distance is higher than the latitude distance in Brant, the number of neurons for longitude axis is higher than the number of neurons for latitude axis after selecting neuron distance value.

**Figure 5:**
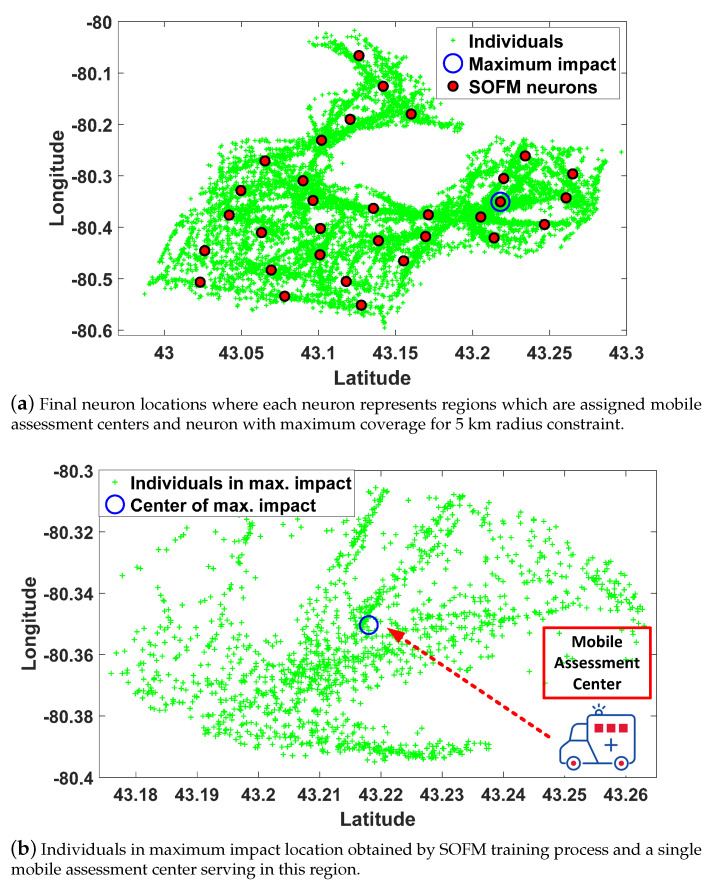
Results of the SOFM algorithm to find all required regions for mobile assessment center assignments in Brant.

Connections are the key factors in the detection of an infected individual in a region which can possibly contribute to community spread by affecting neighborhood regions. Hence, neighborhood districts are considered as high priority regions to isolate all infected cases in these regions. The neuron position providing the maximum individuals under 5 km radius constraint is depicted in Figure 5a. Maximum individuals (i.e., 1566 people) are covered by center of region as depicted in Figure 5b.

**Figure 6:**
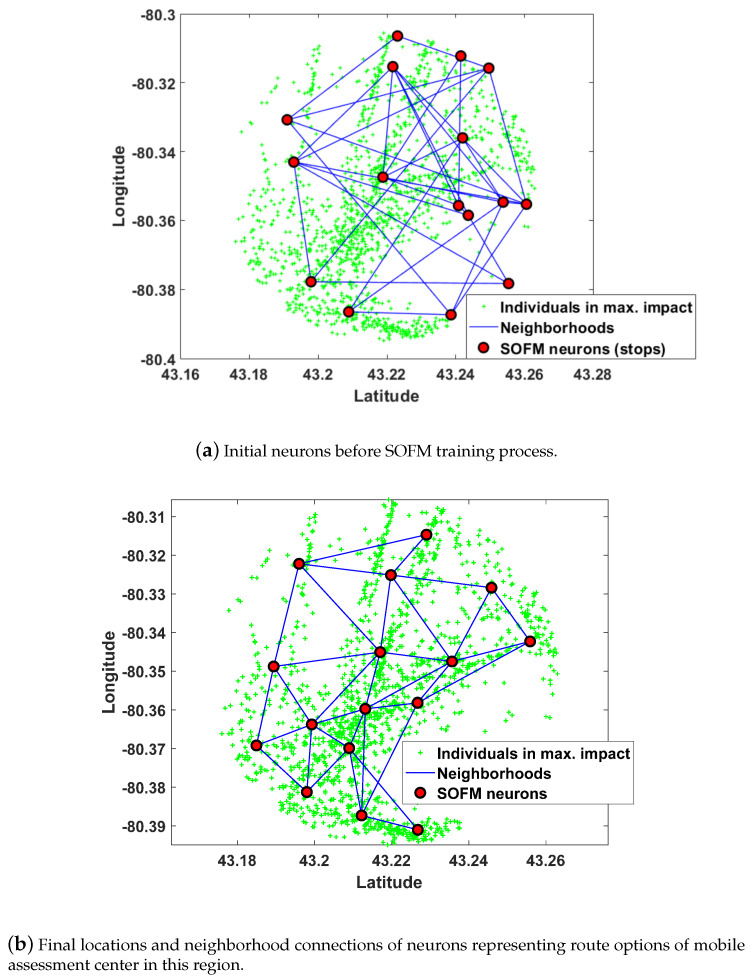
After determining the region by SOFM neurons in Figure 5, the new SOFM provides all stops for route planning of the region in Brant.

Upon determining all regions by SOFM neurons, route planning for the mobile assessment center should be found from beginning to end. SOFM is considered to find all stops to ensure gradual improvement in the individual coverage through efficient updates of neuron positions. SOFM updates not only BMU neuron which is the closest neuron to the individual but also neighborhood neurons connected to the BMU neuron. Therefore, the algorithm ensures maximum coverage in terms of neuron positions. In this phase, the radius constraint is chosen as 400m for evaluating the impact of each stop on the overall coverage. Selecting neuron distance equal to 0.02 offers 16 neurons resulting from 4 (latitude axis) multiplied by 4 (longitude axis) in Figure 6. Initial neuron positions before training and final neuron positions after training are depicted in Figure 6a,b, respectively. Figure 6b can be considered as all possible stops for 16 neurons which are determined by neuron distance equal to 0.02.

**Figure 7:**
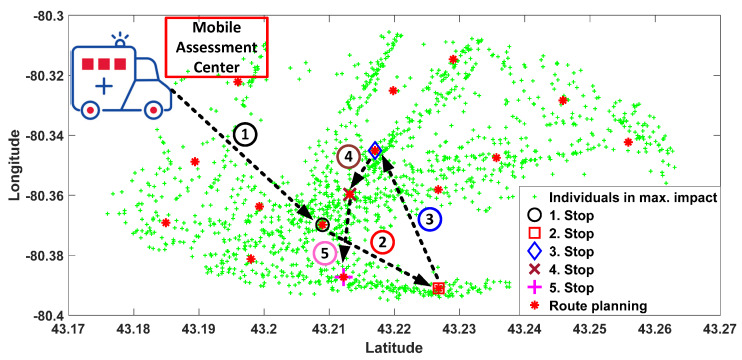
Mobile assessment centers route in region obtained by SOFM neurons for maximum number of individuals of region in Figure 5b in Brant.

**Figure 8:**
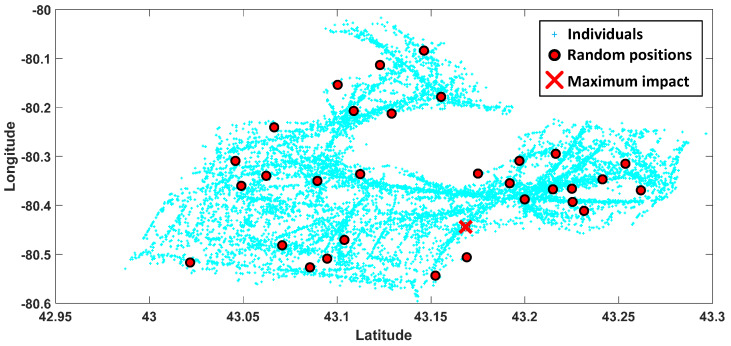
Results of randomly generated locations presenting a regionally assigned mobile assessment center in Brant for individual coverage comparison with SOFM based locations in Figure 5a.

The route planning for 1566 individuals in a 5 km radius of the neuron position which is depicted in Figure 5b is calculated by the SOFM algorithm. In the figure, green points indicate the positions of 1566 individuals in the target regions for a single mobile assessment center. Default values from COVID-19 simulator regarding the distribution of mobile and static individuals are fed into the AI engine to define the classes of individuals with respect to them being mobile or not. The first 5 stops of the total 16 stops which are followed by a mobile assessment center are depicted in Figure 7 in detail. Moreover, random region selection which is required for the performance evaluation of the proposed method (depicted in Figure 8) for Brant. Random region selection is developed by random points generation as similar as SOFM random initialization. Once two times the required number of neurons are utilized at the beginning, then all neurons are sorted from bigger to smaller according to the number of individual coverage. Therefore, random neuron positions are mostly inside the dense region in terms of individuals’ positions.

**Figure 9:**
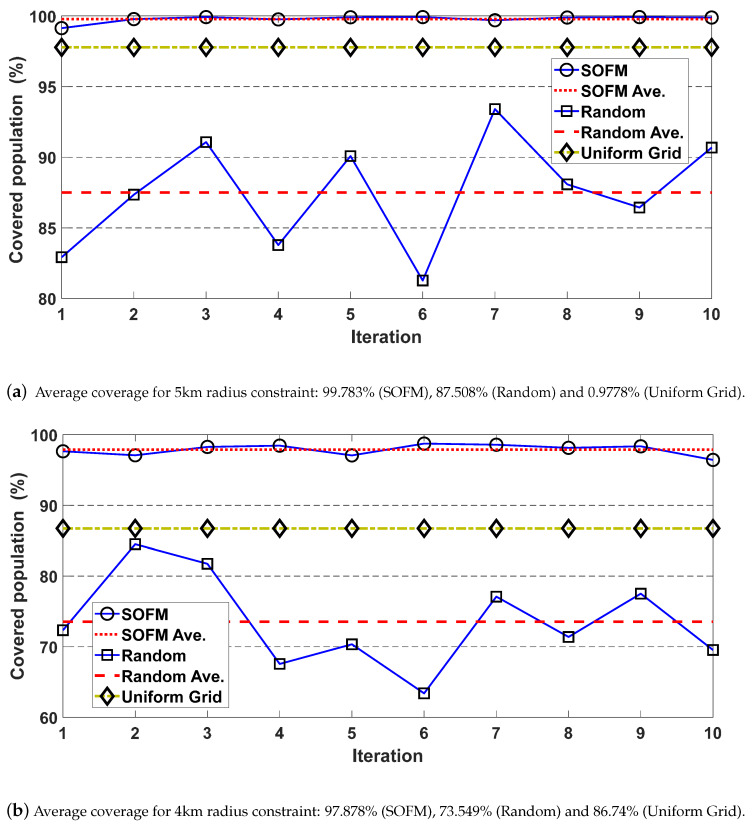
After applying specific radius as the constraint into each region which is defined by SOFM neuron to obtain the individual coverage for SOFM, Random and Uniform grid in Brant

The performance of region selection can be demonstrated by the coverage ratios for both SOFM and random deployment-based methods. Ten runs are used to eliminate the randomness, and the results for 5 km and 4 km radius constraints are depicted in Figure 9. Average coverage values for 5 km constraint are 99.783% for SOFM based approach and 87.508% for random based approach as depicted in Figure 9a, while 97.878% and 73.549% for 4 km radius constraint as depicted in Figure 9b. These results reveal that SOFM-based mobilization does not only lead to lower effects of the epidemic disease on communities but also provides higher coverage than random based region selection.

**Figure 10:**
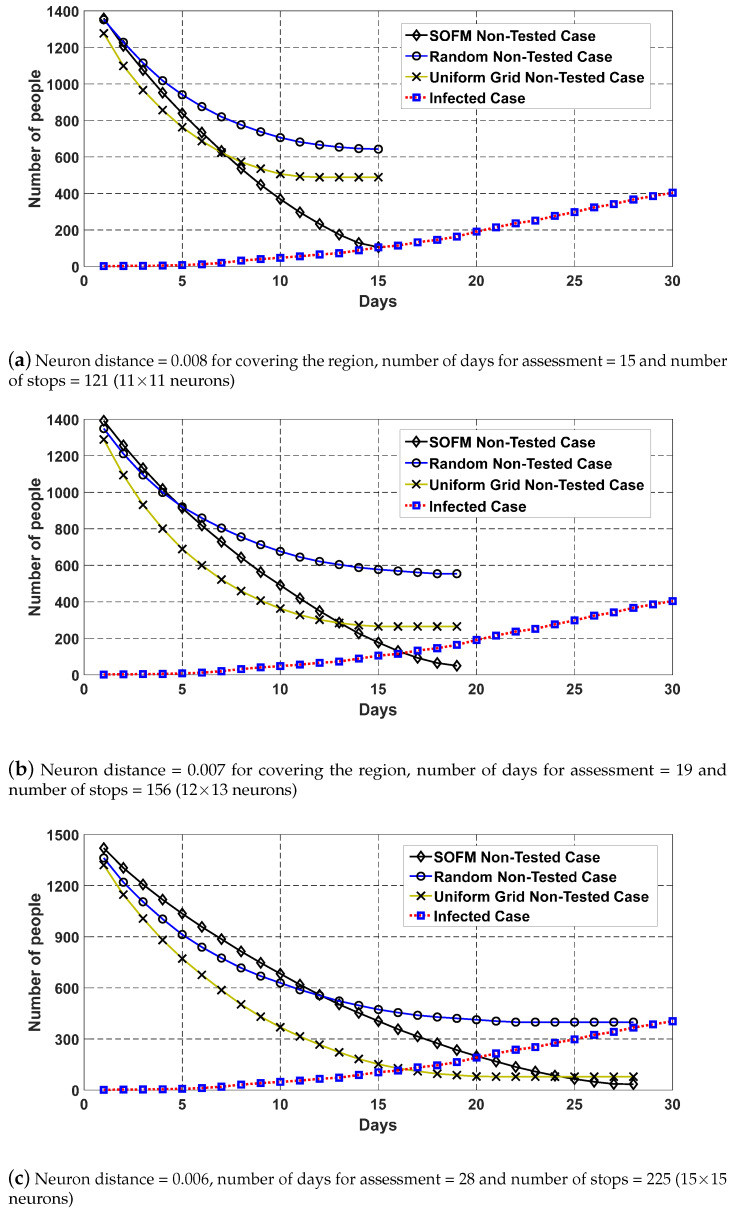
SOFM, Random and Uniform grid based stop generation for a single mobile assessment center of the region covering the maximum number of individuals in Brant. The bigger neuron distance means the lower number of neurons.

As mentioned earlier, COVID-19 simulator is used to generate the number of infected individuals in terms of days so different number of neurons can ensure different worst case scenario regarding with intersection between non-assessed case and infected case as depicted in Figure 10. Figure 10 demonstrates the non-assessed cases against infected cases on the same timeline for the proposed method, random and uniform grid (which is newly added in the revised manuscript). While non-assessed cases are reduced, infected cases keep increasing and the meeting point of both curves indicates the worst-case situation for the elapsed time during the assessment process. In this analysis, if the number of neurons increases, the number of stops and number of days are increased to complete the assessment mission in this region. Moreover, coverage is increased by increasing the number of neurons. However the best result under the worst case analysis requires 121 stops and 15 days as the assessment period as depicted in Figure 10a. Even with less coverage, it is possible to detect at least a single infected case amongst 105 infected individuals on the 15th day. This also proves that a neuron count less than 121 fails the assessment mission for this region. The minimum number of neurons to detect the epidemic/pandemic is varied by the number of individuals and the size of the region.

## 6. Conclusions

COVID-19 like pandemics that are spread at high reproduction rates (e.g., $R_{0}$ being 1.5 to 2.5 in the case of COVID-19) are threatening human lives, healthcare systems and the sustainability of the entire ecosystem on Earth. Recent practice of mitigation adopted by almost all governments reveal the vitality of early-detection of epidemics and pandemics and isolation of confirmed cases. One of the open issues to cope with pandemics is to avoid community spread since a significant portion of confirmed cases remain asymptomatic, which makes effective assessment of communities critical. Under the circumstances, Artificial Intelligence (AI) can play a key role to complement the mitigation efforts. In this article, a self organizing feature map (SOFM)-based mobilization strategy has been proposed to enable mobile assessment agents so to assess the maximum number of individuals in multiple districts of a selected region with minimum possible assessment agents in the shortest possible time. Two classes of analyses have been pursued to verify the efficiency and effectiveness of the proposed methodology. The first class of analyses have focused on maximum coverage in communities where the results have shown that an average 5 km coverage constraint achieves 99.783% coverage outperforming the random deployment of mobile assessment agents by ≈12%. The second class of analyses assume the worst-case scenario, and prove that the minimum number of neurons (i.e., stops) for SOFM (mobile assessment agents) can be achieved on the 15th day following the occurrence of the first confirmed case to be able to detect and isolate all infected individuals whereas the random deployment under the same number of stops over multiple districts fail to detect all cases and leads to non-assessed population remain quadruplicated. This study is currently being extended to run the same experiments on different regions. Furthermore, integration of the existing capacities and time requirements of assessments with the proposed SOFM model is included in our immediate agenda.

## Figures and Tables

**Table 1:** Simulation setup for COVID-19 spreading in a single region.

**Types of Individuals**	**Explanation**
Mobile	Susceptible, on the move
Static	Susceptible, stay at home
Infected	Infects other susceptible individuals within distance "radius"
Immune	Was infected for "duration" steps. No longer infectious
**Controllable Parameters**	**Explanation**
n = 1566	population size
infected = 0.001	initial infected fraction of population
static = 0.20	static individuals ratio
radius = 0.03	infectious distance
duration = 200 days	time of infection
speed = 0.03	spread speed
barrier = 0.0	barrier height

**Table 2:** Simulation results for infected cases in 30 days.

Day	Mobile	Static	Infected	Immune
0	1252	313	1	0
1	1251	313	2	0
2	1250	313	3	0
3	1249	313	4	0
4	1248	313	5	0
5	1246	312	8	0
6	1243	311	12	0
7	1237	309	20	0
8	1228	306	32	0
9	1222	303	41	0
10	1216	302	48	0
11	1209	301	56	0
12	1201	299	66	0
13	1196	297	73	0
14	1181	296	89	0
15	1168	293	105	0
16	1158	293	115	0
17	1145	288	133	0
18	1132	288	146	0
19	1120	282	164	0
20	1098	277	191	0
21	1077	274	215	0
22	1056	273	237	0
23	1043	271	252	0
24	1026	263	277	0
25	1005	263	298	0
26	981	261	324	0
27	966	258	342	0
28	943	256	367	0
29	928	252	386	0
30	914	248	404	0
